# Discovery of Highly Potent Small Molecule Pan-Coronavirus Fusion Inhibitors

**DOI:** 10.3390/v15041001

**Published:** 2023-04-19

**Authors:** Francesca Curreli, Kent Chau, Thanh-Thuy Tran, Isabella Nicolau, Shahad Ahmed, Pujita Das, Christopher D. Hillyer, Mary Premenko-Lanier, Asim K. Debnath

**Affiliations:** 1 Laboratory of Molecular Modeling and Drug Design, Lindsey F. Kimball Research Institute, New York Blood Center, New York, NY 10065, USA; 2 SRI Biosciences (A Division of SRI International), 333 Ravenswood Avenue, Menlo Park, CA 94025, USA; 3 Department of Basic Science, Samuel Merritt University, 3100 Telegraph Avenue, Oakland, CA 94609, USA

**Keywords:** SARS-CoV-2, SARS-CoV, MERS-CoV, Variants of Concern (VoC), pseudoviruses, authentic viruses, ADME

## Abstract

The unprecedented pandemic of COVID-19, caused by a novel coronavirus, SARS-CoV-2, and its highly transmissible variants, led to massive human suffering, death, and economic devastation worldwide. Recently, antibody-evasive SARS-CoV-2 subvariants, BQ and XBB, have been reported. Therefore, the continued development of novel drugs with pan-coronavirus inhibition is critical to treat and prevent infection of COVID-19 and any new pandemics that may emerge. We report the discovery of several highly potent small-molecule inhibitors. One of which, NBCoV63, showed low nM potency against SARS-CoV-2 (IC_50_: 55 nM), SARS-CoV-1 (IC_50_: 59 nM), and MERS-CoV (IC_50_: 75 nM) in pseudovirus-based assays with excellent selectivity indices (SI > 900), suggesting its pan-coronavirus inhibition. NBCoV63 showed equally effective antiviral potency against SARS-CoV-2 mutant (D614G) and several variants of concerns (VOCs) such as B.1.617.2 (Delta), B.1.1.529/BA.1 and BA.4/BA.5 (Omicron), and K417T/E484K/N501Y (Gamma). NBCoV63 also showed similar efficacy profiles to Remdesivir against authentic SARS-CoV-2 (Hong Kong strain) and two of its variants (Delta and Omicron), SARS-CoV-1, and MERS-CoV by plaque reduction in Calu-3 cells. Additionally, we show that NBCoV63 inhibits virus-mediated cell-to-cell fusion in a dose-dependent manner. Furthermore, the absorption, distribution, metabolism, and excretion (ADME) data of NBCoV63 demonstrated drug-like properties.

## 1. Introduction

The devastating effect of coronavirus disease 2019 (COVID-19) caused by the novel SARS-CoV-2, first reported in December 2019 in Wuhan [1], China, continues worldwide. Although two other coronaviruses (CoVs), SARS-CoV-1 in 2003 and MERS-CoV in 2012, were responsible for two severe outbreaks, the current COVID-19 pandemic outpaced all other known pandemics to date. According to data from the Johns Hopkins Coronavirus Resource Center, as of 10 March 2023, there were more than 676 million cases of COVID-19 and 6.7 million deaths globally. In the US, over 103 million cases and more than 1.1 million deaths have been reported (https://coronavirus.jhu.edu/map.html; updates ended on 10 March 2023) [accessed on 17 March 2023].

Although the massive death, hospitalization, uncertainty, and panic have subsided considerably, the continuous emergence of mutant variants with more powerful transmissibility and evading capacity to the currently available vaccines is causing great concern. Furthermore, there have been many reports of “breakthrough” SARS-CoV-2 infections among people who were fully vaccinated [2,3]. In addition, a recent report indicates that about 25% of the world population is hesitant to vaccinate against SARS-CoV-2 [4]. Currently, the FDA approves two small molecule drugs, Veklury (Remdesivir; Gilead, Foster City, CA, USA) and Olumiant (Baricitinib, Lilly, New York, NY, USA). In addition, two small molecule drugs, Paxlovid (a combination of Nirmatrelvir and Ritonavir; Pfizer, New York, NY, USA) and Lagevrio (Molnupiravir; Merck, Rahway, NJ, USA), are approved under an emergency use authorization (EUA). Unfortunately, the resistant mutants of Remdesivir [5,6] and Paxlovid [7] have recently been reported. Furthermore, most approved vaccines and antibody-based therapies show substantial loss of potency against SARS-CoV-2 variants of concern (VOCs) [8,9,10,11]. In addition, the variant (B.1.617.2) [3,12,13], initially identified in India, and the Omicron variants (BA.4 and BA.5) have spread worldwide. David Ho and his team recently reported alarming antibody evasive SARS-CoV-2 subvariants, BQ and XBB [14]. These new variants may pose a substantial challenge to controlling the spread of this virus. Therefore, the continued development of novel drugs having pan-coronavirus inhibition to treat and prevent infection of COVID-19 is urgently needed.

Coronaviruses are positive-sense, enveloped, single-stranded RNA viruses in the family Coronaviridae. The life cycle of these enveloped viruses begins by attaching the trimeric spike surface protein (S) to a host cell [15,16], which is then cleaved into S1 and S2 subunits by furin-like proteases. The S1 subunit of the S protein uses its receptor-binding domain (RBD) to bind to a host-cell receptor, known as the angiotensin-converting enzyme 2 (ACE2) [17,18]. SARS-CoV-1 and SAR-CoV-2 use the same cellular receptor and the cellular transmembrane serine protease 2 (TMPRSS2) for the S protein priming [19,20,21]. The receptor for MERS-CoV is a dipeptidyl peptidase 4 (DPP4; CD26) [22]. The binding of the S1 subunit to a receptor triggers the fusion protein (FP) of the S2 subunit to insert into the cell membrane and help the heptad repeat region 1 (HR1) domain of S2 to form a coiled-coil trimer. This process initiates the binding of the heptad repeat region 2 (HR2) domain of S2 to a hydrophobic groove in the HR1 trimer in an antiparallel manner to form a six-helix bundle (6-HB) structure. The 6-HB formation is typical of other class I membrane fusion proteins in viruses such as influenza, human immunodeficiency virus (HIV), and Ebola [15,23]. This brings the viral and host-cell membranes together for virus-cell fusion [24], a critical step for virus entry into host cells. The fusion mechanism for the S protein of other CoVs, including MERS-CoV, is similar.

The S protein of CoVs is a surface protein exposed on the surface of the mature virus particle. Consequently, it is the primary target for developing neutralizing antibodies and vaccines. The S1 subunit, primarily the RBD domain, and the S2 subunit, especially the HR1 domain, have been targeted for novel drug design [25,26,27,28,29,30,31,32,33]. However, the RBD of the S1 domain is not well conserved among CoVs [34]. As a result, neutralizing antibodies of SARS-CoV-1 show poor cross-reactivity with SARS-CoV-2 [35,36].

Furthermore, the RBD domain of SARS-CoV-2 has been reported to exhibit a higher number of mutations [37,38,39]. Some of these mutations reduce antibodies’ efficacy; hence, the currently available vaccines [40,41]. Recently, BQ.1, BQ.1.1, XBB, and XBB.1 variants of SARS-CoV-2 with multiple mutations on the RBD have been reported, which markedly reduce serum neutralization [14]. Therefore, the RBD may not be an ideal target for developing novel pan-coronavirus inhibitors.

Contrary to the RBD domain, the membrane fusion domains located in the S2 subunit are primarily conserved and, consequently, can be considered as a potential drug target for developing novel small molecule and peptide-based pan-coronavirus inhibitors. These drugs will be critically important in dealing with new pandemics that are certain to emerge in the future.

In this study, we report the discovery of a series of small molecule compounds with highly potent pan-coronavirus activity against SARS-CoV-2, SARS-CoV-1, and MERS-CoV.

## 2. Results and Discussion

### 2.1. Identification of Pan-Coronavirus Inhibitors

An in-depth analysis of the postfusion hairpin structures of CoVs indicated that they were structurally similar and shared critical salt bridges between the HR1 and HR2 regions [30]. For example, K947 of the HR1 domain in SARS-CoV-2 forms a salt bridge with E1182 of the HR2 domain. Similarly, K929 of the HR1 domain in SARS-CoV-1 forms a salt bridge with E1163 of the HR2 domain. Furthermore, a salt bridge between K1021 with E1265 is also present in the MERS-CoV postfusion spike structure [42]. Interestingly, we reported a similar salt bridge interaction between K547 of the N-terminal heptad repeat region (also known as HR1) and D632 of the C-terminal heptad repeat region (also known as HR2) in the HIV-1 gp41 hairpin structure [43]. The later observation motivated us to design a series of highly potent benzoic acid-based inhibitors of HIV-1 gp41 fusion, which contain a COOH group [44]. Based on these remarkable similarities in the formation of salt bridges to form the 6-HB structures and their involvement in virus–cell fusion of HIV-1 gp41 and the CoV S proteins, we hypothesized that COOH-containing inhibitors take part in salt bridge formations by fitting into a cavity in the prefusion trimer structures of CoVs to prevent the 6-HB formation and subsequent virus–cell fusion.

Based on this hypothesis, we screened a set of COOH-containing molecules, which we referred to as NBCoVs. We recently reported their activity against SARS-CoV-2, SARS-CoV-1, and MERS-CoV in spike-pseudotyped antiviral assays [45], showing their pan-coronavirus inhibitory potential. We used the SAR information from that study [45] to search compounds from commercial small molecule drug-like databases. We identified more than 20 molecules which were structurally unique.

### 2.2. Antiviral Activity and Cytotoxicity of the NBCoV Small Molecules in a Pseudovirus Assay

The anti-coronavirus activity of the newly identified NBCoV small molecules was evaluated by infecting two cell types: 293T/ACE2 cells, overexpressing the human receptor ACE2 and A549/ACE2/TMPRSS2 (A549/AT) cells overexpressing both ACE2 and TMPRSS2. Cells were infected with aliquots of the SARS-CoV-2 (USA-WA1/2020) pseudovirus pretreated with escalating concentrations of the NBCoV small molecules for 30 min to calculate the concentration required to inhibit 50% (IC_50_) of SARS-CoV-2 infection. In parallel, the cytotoxicity of the compounds was evaluated in both cell lines to calculate the CC_50_ (the concentration for 50% cytotoxicity) and the selectivity indexes (SI = CC_50_/IC_50_) (Table 1). As shown, compound NBCoV63 had an IC_50_ of 80 ± 7 and 55 ± 3 nM, respectively, and CC_50_ ≥ 50 µM (SI of >625 and 909 for the two cell lines), which was the highest dose we could test for all the compounds due to low solubility. NBCoV35 and NBCoV37, which have a similar structure to NBCoV63, also showed low nanomolar activity in both cell lines (IC_50_ = 66–198 nM). While NBCoV35 had similar toxicity in both cell lines, NBCoV37 induced higher toxicity in 293T/ACE2 cells. NBCoV36 antiviral activity was slightly weaker than the previous three compounds (330 ± 18 and 227 ± 18 nM); in this case as well, we noticed that NBCoV36 exhibited higher toxicity in 293T/ACE2 cells than in A549/AT. NBCoV81 also had antiviral activity with SI > 109 in both cell lines. The remaining compounds had minor to no activity. Based on the results reported in Table 1**,** we decided to validate further the anti-coronavirus activity of the compounds that showed the best SI (NBCoV35-37 and NBCoV63) against SARS-CoV-2 VOCs and other coronaviruses in neutralization assays.

### 2.3. Antiviral Activity of NBCoV35-37 and NBCoV63 against SARS-CoV-2 Mutant (D614G) and Four VOCs

SARS-CoV-2 VOCs carrying multiple mutations in their spike are the cause of concerns due to increased virulence and reduced vaccine efficacy. In this study, we decided to evaluate the effectiveness of our best NBCoV compounds against the first detected virus with a single spike mutation, USA-WA1/2020/D614G, and four VOCs already spread worldwide: Delta B.1.617.2, Omicron B.1.1.529/BA.1, Omicron BA.4/BA.5, and Brazil (Gamma, carrying three spike mutations: K417T/E484K/N501Y) (Table 2). We infected 293T/ACE2 cells and A549/AT cells with the mutant SARS-CoV-2 pseudoviruses in the absence or presence of the NBCoV compounds. We observed that NBCoV63 had potent antiviral activity against all variant pseudoviruses assessed, as indicated by the low IC_50_s detected, in the 34–96 nM range in 293T/ACE2 cells and 26–105 nM in A549/AT cells (Table 2). The best activity of NBCoV35 was recorded against Omicron BA.4/BA.5 in both cell lines (IC_50_s: 94 ± 6 and 170 ± 13 nM, respectively), while it seemed weaker against the Brazil variant (IC_50_s: 506 ± 44 and 292 ± 23 nM). NBCoV37 was also active against all the variants, with IC_50_ values in the 82–267 nM range in 293T/ACE2 cells and 42.5–311 nM in A549/AT cells. Finally, NBCoV36 displayed lower antiviral activity exhibiting a wider range of IC_50_ values (181–1183 nM in 293T/ACE2 cells and 129–332 nM in A549/AT cells). These results suggest that the NBCoV small molecules maintain their antiviral activity against the SARS-CoV-2 D614G-mutant and other VOCs.

To evaluate these small molecules as pan-coronavirus inhibitors, we verified their activity against SARS-CoV (Table 3) and MERS-CoV (Table 4) pseudoviruses. We found that NBCoV63 was again the most potent compound in both cell lines infected with SARS-CoV (Table 3), with calculated IC_50_s of 110 ± 0.5 nM (SI: 455) in 293T/ACE2 cells and 59 ± 1.5 nM (SI: 848) in A549/AT cells. NBCoV35-37 also maintained their inhibitory activity against this virus with IC_50_s in the range of 340–660 nM (SI: 65–132) in 293T/ACE2 cells and 95–363 nM (SI: 138–526) in A549/AT cells.

Furthermore, as reported in Table 4, yet again, NBCoV63 was the most potent compound against the MERS-CoV pseudovirus in both cell lines (IC_50_: 75 nM in Caco-2 cells, IC_50_: 102 nM in MRC-5 cells, and SIs > 667 and >490, respectively). NBCoV37 also had a potent MERS-CoV inhibitory activity with IC_50_ in the range 161–229 nM, while NBCoV35-36 had slightly weaker activity against this virus in both cell lines (IC_50_ 428–837). The data suggest that NBCoV35-37 and NBCoV63 indeed possess pan-coronavirus activity.

To validate our findings, we measured the activity of the NBCoV compounds in Calu-3 cells, a human lung cell line. These non-small-cell lung cancer cells are permissive to SARS-CoV-2 strains, SARS-COV-1, and MERS-CoV virus strains. We noticed that NBCoV63 maintained its excellent antiviral activity against all the viral clones evaluated, with IC_50_ of 120–220 nM (Table 5). On the contrary, NBCoV35-37 showed higher IC_50_s in these cells than those detected with the cell lines described above (Table 1, Table 2, Table 3 and Table 4). However, they maintained good antiviral activity against the omicron BA.4/BA.5 variants. The CC_50_ in these cells suggested no interference from toxicity.

Finally, to verify the specificity of the NBCoV compounds for the coronaviruses, we evaluated them against the amphotropic murine leukemia virus (A-MLV), which enters the cells via macropinocytosis [46]. We found that while NBCoV35-37 had insignificant activity against this virus, however, NBCoV63 IC_50_ was about 3.8-fold higher than the IC_50_ value detected against SARS-CoV-2 in the same cell line (Table 6). Although further investigation will be necessary to explain this finding, we can exclude unspecific activity against the cells as the CC_50_ was >50 µM. Additionally, we performed two sets of experiments. In the first one, we exposed A549/AT cells to the compounds for 30 min before infection with SARS-CoV-2, and in the second experiment, we added the virus and the compounds to the cells simultaneously. We found no toxicity and no activity of the compounds against SARS-CoV-2 in both experiments (data not shown). These findings confirmed that the NBCoV compounds target the viruses to inhibit viral entry and do not affect the cell well-being as they did not induce cell toxicity and did not prevent cell infection when the cells were pretreated with the compounds, but they only inhibit viral infection when the virus was pretreated before adding it to the cells.

### 2.4. NBCoV63 Inhibited Three Variants of the Replication-Competent Authentic Virus SARS-CoV-2 and Two Closely Related Highly Pathogenic Coronaviruses, SARS-CoV-1 and MERS-CoV

Following the demonstration of the antiviral activity of NBCoV63 against mutant SARS-CoV-2 and VOCs SARS-CoV-1 and MERS in pseudovirus assays, we next selected this compound as the best inhibitor and lead compound. We evaluated its antiviral activity using a traditional plaque-reduction assay against live SARS-CoV-2 variants. Three variants were selected: SARS-CoV-2 Hong Kong/VM20001061/2020 (tested at an 0.5 MOI), SARS-CoV-2 Omicron hCoV-19/USA/NY-MSHSPSP-PV56475/2022, and SARS-CoV-2 Delta hCoV-19/USA/PHC658/2021 (both tested at 0.01 MOI). The control compound Remdesivir had an IC_50_ of 3.44 and 2.82 µM against the parent HK strain and the Omicron variant, respectively (Table 7), and an IC_50_ of 12.5 µM against the Delta variant. NBCoV63 had IC_50_ values that were lower for Delta and HK but slightly higher for the Omicron variant. Furthermore, NBCoV63 was evaluated against SARS-CoV-1 Urbani and MERS-CoV; once again we found that its IC_50_ was slightly higher than the IC_50_ of Remdesivir against SARS-CoV-1 but more than half lower against MERS-CoV. Moreover, there was no toxicity in Calu-3 cells at the dilutions tested. These data demonstrate that NBCoV63 has similar antiviral activity as Remdesivir against live SARS-CoV-2 viruses, SARS-CoV-1, and MERS-CoV. The data confirm that NBCo63 has comparable antiviral potency to Remdesivir. Photos of the plaque-reduction assays for NBCoV63 and Remdesivir are shown in the supplemental information (Figure S1).

### 2.5. NBCoV Small Molecules Inhibited the SARS-CoV-2 Mediated Cell-to-Cell Fusion

**Figure 1:**
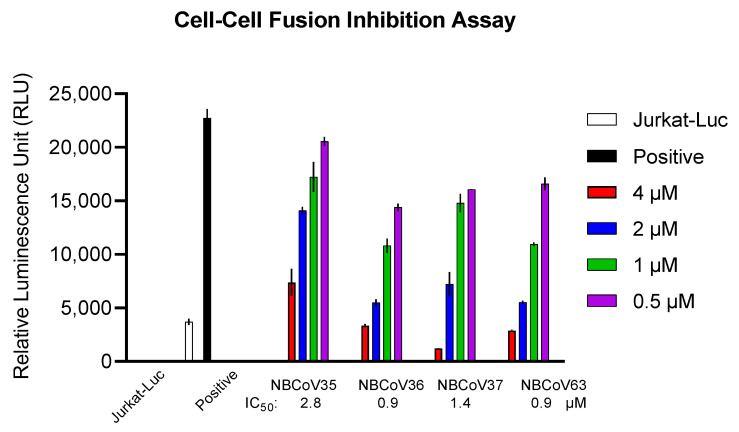
SARS-CoV-2-mediated cell-to-cell fusion inhibition assay. Jurkat cells expressing the luciferase gene and the SARS-CoV-2 full spike wild-type gene from Wuhan-Hu-1 isolate were used as donor cells and the 293T/ACE2 as acceptor cells. Positive represents 293T/ACE2 cells cocultured with Jurkat cells in the absence of NBCoVs. Jurkat-Luc represents 293T/ACE2 cells cocultured with Jurkat cells expressing the luciferase gene only in the absence of NBCoVs. Columns represent the means plus/minus standard deviations (n = 2–4).

It has been reported that cell-to-cell direct contact/fusion is another efficient viral spreading mode [47]. It facilitates the infection of adjacent cells without producing free viruses and, at the same time, contributes to tissue damage and syncytia formation. We used a recently described cell-to-cell fusion inhibition assay [45] to evaluate the inhibitory activity of four concentrations of NBCoV 35–37 and NBCoV63 on SARS-CoV-2 mediated cell-to-cell fusion (Figure 1). This assay uses Jurkat cells expressing a luciferase gene and the wild-type S gene from the SARS-CoV-2 Wuhan-Hu-1 isolate as donor cells and 293T/ACE2 cells as acceptor cells [45]. As shown in Figure 1, all the NBCoV compounds inhibited cell-to-cell fusion in a dose-dependent manner. NBCoV63 and NBCoV36 were the most effective compounds in this assay, inducing 50% inhibition at 0.9 µM. NBCoV37 was slightly less effective with an IC_50_ of 1.4 µM while NBCoV35 was the least active. Taken together these data suggest that the NBCoV compounds not only disrupt the binding of SARS-CoV-2 S protein with the receptor but also interfere with the virus-mediated cell-to-cell fusion.

### 2.6. Computer-Based Determination of the Binding Site of NBCoV63 in the SARS-CoV-2 Spike Protein

**Figure 2:**
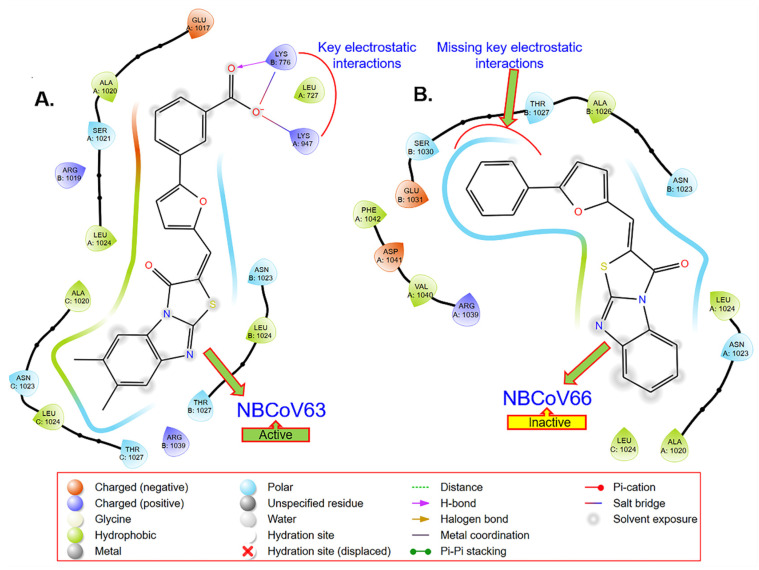
GLIDE-based docking of an active and an inactive NBCoV demonstrates that (**A**) the COOH group of the active compound (NBCoV63) has the key salt-bridge interactions; (**B**) these key interactions are missing.

In the absence of any information about the structures of the inhibitors bound to the SARS-CoV-2 S protein, we performed a GLIDE-based docking study (Schrodinger, CA, USA) using the SARS-CoV-2 prefusion structure of the S protein (PDB: 6VSB) [48] and our knowledge of the HIV-1 gp41 fusion protein [49,50,51,52,53]. We were intrigued to find that NBCoV63, one of the most active inhibitors in the SARS-CoV-2 pseudovirus assay, scored the best in the docking study (Figure 2A). Most strikingly, we observed that the COOH moiety of NBCoV63 formed a salt bridge/H-bond with K947 of the A chain of the HR1 domain of the S protein, which was previously shown to form a salt bridge with E1182 of the HR2 domain in the postfusion hairpin structure of the S protein of SARS-CoV-2. Furthermore, we found that K776 of the B chain of the HR1 domain created similar interactions. Figure 2B confirms the importance of the meta COOH group in the antiviral activity of this class of compounds. NBCoV66, which has all the critical scaffold in its structure but is lacking the COOH group on the meta position of the phenyl group, showed no antiviral activity. The docking simulations also indicated that the phenyl part of the tricyclic structure is not making any significant contact with the spike protein. These observations are expected to help in the optimization of this class of inhibitors. As a follow-up of the docking-based observations, we plan to confirm the binding of NBCoV63 and others by direct binding by surface plasmon resonance (SPR) using the prefusion SARS-CoV-2 spike protein as we reported earlier [45].

### 2.7. In Vitro ADME

Assessment of ADME properties in the early stage of drug discovery and development is necessary to reduce drug failure in later stages of drug development [54]. We decided to study the ADME properties of our best inhibitor, NBCoV63, which had potent pan-coronavirus activity, low cytotoxicity, and excellent SI. The ADME properties of NBCoV63 were evaluated using Cyprotex US, LLC (Watertown, MA, USA). Solubility is one of the essential properties of a drug and plays a critical role in drug discovery. The data in Table 8 indicate that the solubility of NBCoV63 is low. Therefore, special attention should be given to increasing its solubility during its optimization phase. One option will be to attach a solubilizing group in a non-pharmacophoric site, which is expected to increase its solubility. The apparent permeabilities of NBCoV63 in the Caco-2 permeability assay are also low. This assay is used to understand oral drugs’ gastrointestinal (GI) absorption. The data also indicate that P-glycoprotein (P-gp)-mediated efflux is involved. The primary role of P-gp is to protect the body from harmful substances by removing drugs absorbed in the intestines back into the gut lumen. We examined the metabolic stability of NBCoV63 in the human liver microsome because the liver is the most important site of drug metabolism in the body. The clearance data (Cl_int_) in Table 8 indicate that NBCoV63 is a low-clearance compound with a half-life of >180 min. It is worthwhile to mention that low clearance is often a goal in drug development because it allows for lower doses to be used, which reduces drug-related toxicities. The binding data for NBCoV63 in human plasma showed that the inhibitor is >99.5% bound (Table 8), which is high, although many drugs have >98% plasma protein binding, high protein binding does not affect the success of drug candidates. The misconception that high plasma protein binding is a problem for drug candidates was elegantly discussed by Smith et al. in 2010 [55]. To identify potential drug–drug interactions, we evaluated NBCoV63 against six CYP450 isoforms (CYP1A2, CYP2B6, CYP2C8, CYP2C9, CYP2C19, CYP2D6) that play an essential role in metabolizing almost 80% of all drugs in the human body [56,57].

The following guideline is usually used for the CYP inhibition assessment [58]:

IC50 > 10 µM (CYP inhibition low)

<10 µM (CYP inhibition moderate)

<3 µM (CYP inhibition high)

This classification indicated that NBCoV63 had no inhibition up to 25 µM (Table 8, indicating high tolerance to CYP-mediated metabolism.

## 3. Conclusions

We identified a series of small molecule benzoic acid analogs that showed highly potent pan-coronavirus activity in a pseudovirus-based single-cycle assay. These inhibitors also showed low cytotoxicity, therefore, exhibiting high SI values. One of them, NBCoV63, showed the most consistent high potency against SARS-CoV-2, their mutants, and VOCs, SARS-CoV-1, and MERS-CoV in different cell lines. Most significantly, NBCoV63 also showed similar efficacy profiles to Remdesivir against an authentic SARS-CoV-2 (Hong Kong strain), its Delta variant, and against MERS-CoV; however, Remdesivir showed about 2–3-fold better activity against the Omicron variant and against SARS-CoV-1 in a plaque assay in Calu-3 cells. In addition, we showed that NBCoV63 inhibited virus-mediated cell-to-cell fusion in a dose-dependent manner. The in vitro ADME data of NBCoV63 shows drug-like properties. However, the solubility and GI absorption of this class of inhibitors needs to be improved. Nevertheless, NBCoV63 can be considered a lead candidate for medicinal chemistry-based optimization.

## 4. Experimental Section

### 4.1. Cells and Plasmids

The MRC-5 (fibroblasts cell line isolated from lung tissue), Caco-2 (epithelial cell line isolated from colon tissue), Calu-3 (epithelial cell line isolated from lung tissue), and HEK293T/17 cells were purchased from ATCC (Manassas, VA, USA). The human lung carcinoma A549 cells expressing human ACE2 and TMPRSS2 and the spike pseudotyping vectors Delta Variant (B.1.617.2) pLV-SpikeV8, Omicron Variant (B.1.1.529/BA.1) pLV-SpikeV11, and Omicron Variants (BA.4/BA.5) pLV-SpikeV13 were purchased from InvivoGen (San Diego, CA, USA). The human T-Cell lymphoma Jurkat (E6-1) cells were obtained through the NIH ARP. The 293T/ACE2 cells and the two plasmids pNL4-3^∆Env^-NanoLuc and pSARS-CoV-2-S^Δ19^ were kindly provided by Dr. P.Bieniasz of Rockefeller University [59]. The pSV-A-MLV-Env (envelope) expression vector [60,61] and the Env-deleted proviral backbone plasmids pNL4-3.Luc.R-E- DNA [62,63] were obtained through the NIH ARP. The two plasmids, pSARS-CoV and pMERS-Cov, were kindly provided by Dr. L. Du of the New York Blood Center. The vector expressing the SARS-CoV-2 full spike wild-type (WT) gene from the Wuhan-Hu-1 isolate (pUNO1-SARS-S) was purchased from InvivoGen (San Diego, CA, USA). The pFB-Luc plasmid vector was purchased from Agilent Technologies (Santa Clara, CA, USA).

### 4.2. Small Molecules

We screened a set of twenty-two small molecules, of which NBCoV35-37 were purchased from Chembridge Corporation (San Diego, CA, USA) and NBCoV62-82 were purchased from ChemDiv, Inc. (San Diego, CA, USA). The purity of all purchased compounds is >90%. Details of the analyses are reported in the supporting information.

### 4.3. Pseudoviruses Preparation

Pseudoviruses capable of single-cycle infection were prepared by transfecting 8 × 10^6^ HEK293T/17 cells with a proviral backbone plasmid and an envelope expression vector by using FuGENE HD (Promega, Madison, WI, USA) and following the manufacturer’s instructions as previously described [45]. We used the HIV-1 Env-deleted proviral backbone plasmid pNL4-3^∆Env^-NanoLuc DNA and the Envs SARS-CoV-2 and its VOCs, SARS-CoV and the MERS-CoV, to obtain the respective pseudoviruses. For the A-MLV pseudovirus, we used the Env-deleted proviral backbone plasmids pNL4-3.Luc.R-.E- DNA and the pSV-A-MLV-Env expression vector. Pseudovirus-containing supernatants were collected two days after transfection, filtered, tittered, and stored in aliquots at −80 °C. Pseudovirus titers were determined with the Spearman–Karber method [64] to identify the 50% tissue culture infectious dose (TCID_50_) by infecting the different cell types as previously described [45]. The incorporation of the respective spike proteins into the pseudoviruses was confirmed as previously described [45].

### 4.4. Measurement of Antiviral Activity

The antiviral activity of the NBCoV small molecules was evaluated in a single-cycle infection assay by infecting different cell types with the SARS-CoV-2 and its VOCs, SARS-CoV or MERS-CoV pseudoviruses, as previously described [32,45,65].

**A549/AT cells.** We evaluated the antiviral activity by preincubating aliquots of the pseudovirus SARS-CoV-2, its VOCs, and SARS-CoV (1500 TCID_50_/well and at an MOI of 0.1) with the escalating concentrations of the NBCoVs for 30 min before adding to the A549/AT cells (1 × 10^4^ cells/well in a 96-well cell culture plate). A549/AT cells cultured with medium with or without pseudoviruses were included as positive and negative controls, respectively. Following 48 h incubation, the cells were washed with PBS and lysed with 50 µL of lysis buffer (Promega). Twenty-five µL of the lysates were transferred to a white plate and mixed with the same volume of Nano-Glo^®^ Luciferase reagent. The luciferase activity was measured with the Tecan SPARK. The percent inhibition and the IC_50_ (the half-maximal inhibitory concentration) values were calculated using the GraphPad Prism 9.0 software (San Diego, CA, USA).

**293T/ACE2 cells.** The 96-well plates used for the 293T/ACE2 cells were coated with 50 µL of poly-l-lysine (Sigma-Aldrich, St. Louis, MO, USA) at 50 µg/mL as previously described [45]. The neutralization assay and the data collection were performed as described above for A549/AT cells.

**Calu-3 cells**. The antiviral activity of the small molecules in Calu-3 cells was evaluated as reported above by plating 5 × 10^4^ cells/well the cells in a 96-well cell culture plate and incubating overnight. On the following day, aliquots of the pseudoviruses at an MOI of 0.1 were preincubated with escalating concentrations of the NBCoV small molecules for 30 min then added to the cells. The assay and data collection were performed as described above.

**Caco-2 and MRC-5 cells**. Caco-2 and MRC-5 cells were plated at 1 × 10^4^ cells/well in a 96-well cell culture plate and incubated overnight. On the following day, aliquots of the MERS-CoV pseudovirus at about 1500 TCID_50_/well at an MOI of 0.1 were pretreated with graded concentrations of the small molecules for 30 min and added to the cells. The assay and data collection were performed as described above.

**Plaque-reduction assay.** We evaluated NBCoV63 and the control article Remdesivir by using a traditional plaque-reduction assay. The assay was performed in a 24-well plate using Calu-3 cells. The cells were seeded 24 h before the assay at a density to reach confluency. The test compound and the control article were diluted 1:2 at a 2× concentration in a 96-well plate in duplicate. The final test compound concentration range was from 0.04 µM to 20 µM, and the final control article concentrations range was from 0.1 µM to 50 µM. The compounds were tested against the following viruses: SARS-CoV-2 Hong Kong/VM20001061/2020 (HK) at 0.5 MOI; SARS-CoV-2 Omicron hCoV-19/USA/NY-MSHSPSP-PV56475/2022 and SARS-CoV-2 Delta hCoV-19/USA/PHC658/2021 at 0.01 MOI; SARS-CoV-1 Urbani (obtained from BEI resources) at 0.02 MOI. MERS-CoV was obtained from BEI resources (MERS-CoV, EMC/2012, NR-44260) and tested at 0.0001 MOI. An equal volume of the diluted virus was added to each well to make the final compound concentration 1×. Twenty-four well cell seeding at 240,000 cells/well was used for MOI calculations. All incubations were conducted in a 37 °C and 5% CO_2_ incubator in an approved BSL3 laboratory. The virus and compound dilutions were gently mixed and incubated for 1 h. Following incubation, the virus and diluted compound mixtures were transferred to the respective wells of the Calu-3 cells. The mixture was incubated with the Calu-3 cells for 1 h with gently rocking every 15 min. Following incubation, 500 µL of a 1:1 2× medium and 1% agarose was added to each well and incubated. The assays for SARS-CoV-2 and SARS-CoV-1 ran for 72 h, and the assay for MERS-CoV ran for 48 h. After incubation, 0.2 mL of 10% formalin was added to the agarose plug for at least 1 h. The agarose plug was removed, and the cells stained in 0.2 mL of a 1% crystal violet in 10% formalin mixture and incubated for 15 min. The wells were washed twice with PBS (500 µL/well/wash). Plaques were visualized and % virus inhibition recorded for each well. Compounds without virus were tested for toxicity using the same procedure, and CC50 was calculated. Prism software was used for all calculations.

### 4.5. Evaluation of Cytotoxicity

The cytotoxicity of NBCoV small molecules in the different cell types was performed in parallel with the neutralization assay and evaluated using the colorimetric CellTiter 96^®^ AQueous One Solution Cell Proliferation Assay (MTS) (Promega, Madison, WI, USA) following the manufacturer’s instructions. Briefly, for the cytotoxicity assay in A549/AT, 293T/ACE2, Caco-2, and MRC-5 cells, 1 × 10^4^/well cells were plated in a 96-well cell culture plate and incubated overnight; for the same assay in Calu-3 cells, we used 5 × 10^4^/well cells. The following day, aliquots of escalating concentrations of the NBCoV compounds were added to the cells and incubated at 37 °C. Following 48 h incubation, the MTS reagent was added to the cells and incubated for 4 h at 37 °C. The absorbance was recorded at 490 nm. The percentage of cytotoxicity and the CC_50_ values were calculated using the GraphPad Prism 9.0 software.

### 4.6. Drug Sensitivity of Spike-Mutated Pseudovirus

Amino acid substitutions or deletions were introduced into the pSARS-CoV-2-S^Δ19^ expression vector by site-directed mutagenesis (Stratagene, La Jolla, CA, USA) by following the manufacturer’s instructions and using mutagenic oligonucleotides SaCoV2-K417T-F: GCTCCAGGGCAAACTGGAACGATTGCTGATTATAATTAT and SaCoV2-K417T-REV: ATAATTATAATCAGCAATCGTTCCAGTTTGCCCTGGAGC. Primers for mutations E484K, N501Y, and D614G have already been described [45]. Site mutations were verified by sequencing the entire spike gene of each construct. To obtain the SARS-CoV-2 pseudovirus carrying the amino acid substitutions, HEK293T/17 cells were transfected with the HIV-1 Env-deleted proviral backbone plasmid pNL4-3^∆Env^-NanoLuc DNA and the mutant pSARS-CoV-2-S^Δ19^ as described above. Pseudoviruses were tittered by infecting 293T/ACE2 and A549/AT cells as described above. The neutralization assay in A549/AT and 293T/ACE2 cells was performed as described above.

### 4.7. Cell-to-Cell Fusion Inhibition Assay

For the SARS-CoV-2 mediated cell-to-cell fusion assay, we used Jurkat cells which transiently expressed the luciferase gene and stably expressed the SARS-CoV-2 full spike wild-type (WT) gene from the Wuhan-Hu-1 isolate as donor cells and the 293T/ACE2 as acceptor cells as previously described [45]. Briefly, Jurkat cells were transfected with the SARS-CoV-2 WT expression vector by using FuGene HD and following the manufacturer’s instructions. Following 24 h incubation, the cells were washed and selected for the SARS-CoV-2 spike expression using Blasticidin at 10 µg/mL concentration. In parallel, un-transfected control Jurkat cells were exposed to the same concentration of Blasticidin to rule out cell resistance to the antibiotic; the culture of control cells was entirely depleted in about 14 days. The antibiotic was replaced every four days, and the selection lasted for about 20 days. On the day before the assay, the 293T/ACE2 were plated in a 96-well cell culture plate at 8 × 10^4^/well, while the Jurkat cells were transfected with the pFB-Luc expression plasmid DNA. Following 20 h incubation, the Jurkat cells at 8 × 10^4^/well were incubated with four escalating concentrations of the NBCoV compounds for 1 h, then transferred to the respective wells containing the 293T/ACE2 cells. 293T/ACE2 cells cultured with or without Jurkat cells were included as positive and negative controls, respectively. As an additional control, a set of 293T/ACE2 cells were incubated with Jurkat cells expressing the luciferase gene only (Jurkat-Luc). The plate was spun for 5 min at 1500 rpm and then incubated for 4 h at 37 °C. The wells were carefully washed twice with 200 µL of PBS to remove the Jurkat cells that did not fuse with the 293T/ACE2 cells. Finally, the cells were lysed to immediately measure the luciferase activity relative luminescence unit (RLU).

### 4.8. GLIDE-Based Docking

We used the automated docking software GLIDE in Schrödinger Suit 2022-4 (Schrödinger, LLC, NY, USA), which uses a hierarchical series of filters in searching for appropriate ligand conformation in the active site of a target protein. It uses a funnel-shaped scoring process to sort out the best conformations and orientations of the ligand (defined as pose) based on its interactions with the receptor. GLIDE has been used successfully in drug design.

We used the SARS-CoV-2 prefusion structure of the S protein (PDB: 6VSB) as the target protein. We used the “Protein Preparation Wizzard” within Maestro to optimize hydrogens, bond orders, charges, and steric clashes using the OPLS3e force field. We used this optimized protein structure to create a grid file encompassing the area surrounding K947, which was previously shown to form a salt bridge with E1182 of the HR2 domain in the postfusion hairpin structure of the S protein of SARS-CoV-2 [30].

We generated three-dimensional coordinates of the ligands and their isomeric, ionization, and tautomeric states using the Ligand Preparation Wizard (including Ionizer) within the Schrödinger Suite 2022-4. The conformational flexibility of the ligands was handled via an exhaustive conformational search. Initially, we used Schrödinger’s proprietary GlideScore scoring function in extra precision (XP) mode to score the optimized poses.

**In Vitro ADME Study.** Details of the in vitro ADME study and data analyses can be found in the Supplemental.

## Figures and Tables

**Table 1:** Screening of the neutralization activity of NBCoV compounds against NL4-3^ΔEnv^-NanoLuc/SARS-CoV-2 (USA-WA1/2020) pseudovirus (IC_50_), toxicity evaluation (CC_50_), and selectivity indexes (SI) in two cell lines.

Compounds	Structure	293T/ACE2 Cells			A549/ACE2/TMPRSS2 Cells		
		IC_50_ (nM) ^a^	CC_50_ (µM) ^a^	SI	IC_50_ (nM) ^a^	CC_50_ (µM) ^a^	SI
NBCoV35	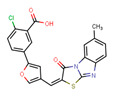	198 ± 9	~50	253	126 ± 3	~50	397
NBCoV36	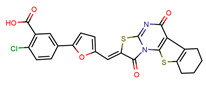	330 ± 18	43 ± 0.9	130	227 ± 18	>50	>220
NBCoV37	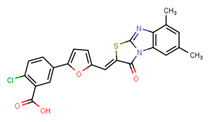	66 ± 2	42.5 ± 2	644	145 ± 12	~50	345
NBCoV62	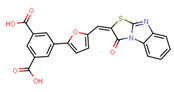	>10,000	>50	ND	>10,000	>50	ND
NBCoV63	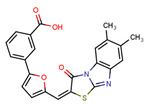	80 ± 7	>50	>625	55 ± 3	~50	909
NBCoV65	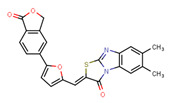	>10,000	>50	ND	>10,000	>50	ND
NBCoV66	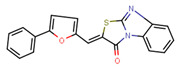	>10,000	>50	ND	>10,000	>50	ND
NBCoV68	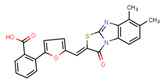	3900 ± 100	>50	>13	2300 ± 70	>50	>22
NBCoV69	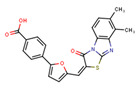	2550 ± 105	>50	>20	3000 ± 400	>50	>17
NBCoV70	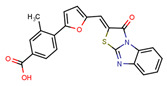	9500 ± 1000	>50	>5	>10,000	>50	ND
NBCoV71	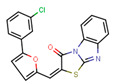	>10,000	48 ± 2	ND	>10,000	40 ± 2	ND
NBCoV72	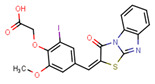	>10,000	>50	ND	>10,000	>50	ND
NBCoV73	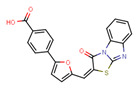	>10,000	>50	ND	>10,000	>50	ND
NBCoV74	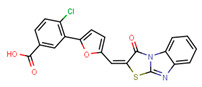	>10,000	>50	ND	>10,000	>50	ND
NBCoV75	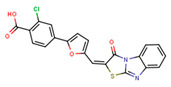	5200 ± 600	>50	>10	3300 ± 700	>50	>15
NBCoV76	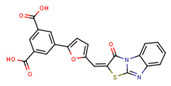	>10,000	>50	ND	>10,000	>50	ND
NBCoV77	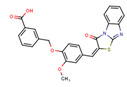	>10,000	>50	ND	>10,000	>50	ND
NBCoV78	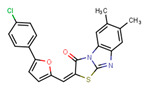	>10,000	>50	ND	8100 ± 200	>50	>6
NBCoV79	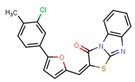	>10,000	>50	ND	>10,000	>50	ND
NBCoV80	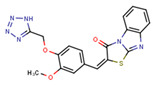	>10,000	>50	ND	>10,000	>50	ND
NBCoV81	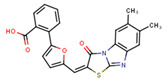	387 ± 64	>50	>129	459 ± 23	>50	>109
NBCoV82	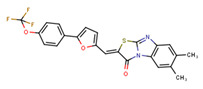	>10,000	>50	ND	>10,000	>50	ND

^a^ The reported IC_50_ and CC_50_ values represent the means ± standard deviations (n = 3). ND = Not Determined.

**Table 2:** Antiviral activity of the NBCoV small molecules in the single-cycle assay in two cell lines against pseudovirus NL4-3^ΔEnv^-NanoLuc/SARS-CoV-2 mutant (D614G) and its variants of concern (VOC) (IC_50_).

Compounds	SARS-CoV-2 IC_50_ (nM) ^a^				
	USA-WA1/2020 D614G	Variants of Concern (VOC)			
		Delta (B.1.617.2)	Omicron (B.1.1.529/BA.1)	Omicron (BA.4/BA.5)	Brazil (Gamma: K417T/E484K/N501Y)
**293T/ACE2 Cells**					
NBCoV35	195 ± 10	335 ± 5	173 ± 30	94 ± 6	506 ± 44
NBCoV36	430 ± 31	802 ± 143	368 ± 90	181 ± 12	1183 ± 58
NBCoV37	157 ± 1.5	267 ± 23	135 ± 3	82 ± 8	260 ± 52
NBCoV63	40.8 ± 1.2	84 ± 0.5	96 ± 11	34 ± 1	77.5 ± 17.3
**A549/ACE2/TMPRSS2 Cells**					
NBCoV35	266 ± 15	236 ± 6	201 ± 38	170 ± 13	292 ± 23
NBCoV36	150 ± 2	332 ± 26	129 ± 2	310 ± 21	149 ± 26
NBCoV37	311 ± 33	130 ± 35	42.5 ± 1	215 ± 10	73 ± 14
NBCoV63	42.5 ± 1	105 ± 1	60 ± 3	26 ± 0.5	45.5 ± 2

^a^ The reported IC_50_ values represent the means ± standard deviations (n = 3).

**Table 3:** Antiviral activity of the NBCoV small molecules in a single-cycle assay in two cell lines against pseudovirus NL4-3^ΔEnv^-NanoLuc/SARS-CoV-1 (IC_50_) and SI.

	SARS-CoV-1			
Compound	293T/ACE2 Cells		A549/ACE2/TMPRSS2 Cells	
	IC_50_ (nM) *	SI	IC_50_ (nM) *	SI
NBCoV35	380 ± 69	132	363 ± 11	138
NBCoV36	660 ± 30	65	147 ± 19	340
NBCoV37	340 ± 34	125	95 ± 1	526
NBCoV63	110 ± 0.5	455	59 ± 1.5	848

* The reported IC_50_ values represent the means ± standard deviations (n = 3).

**Table 4:** Antiviral activity of the NBCoV small molecules in the single-cycle assay in two cell lines against pseudovirus NL4-3^ΔEnv^-NanoLuc/MERS-CoV (IC_50_), toxicity (CC_50_), and SI.

Compound	MERS-CoV					
	Caco-2 Cells			MRC-5 Cells		
	IC_50_ (nM) ^a^	CC_50_ (µM) ^a^	SI	IC_50_ (nM) ^a^	CC_50_ (µM) ^a^	SI
NBCoV35	472 ± 43	>50	>106	715 ± 43	>50	>70
NBCoV36	428 ± 34	>50	>117	837 ± 38	>50	>60
NBCoV37	161 ± 53	>50	>311	229 ± 20	48 ± 2	210
NBCoV63	75 ± 25	>50	>667	102 ± 1	>50	>490

^a^ The reported IC_50_ and CC_50_ values represent the means ± standard deviations (n = 3).

**Table 5:** Antiviral activity of the NBCoV small molecules in the single-cycle assay in Calu-3 cells against pseudovirus SARS-CoV-2 (USA-WA1/2020) and its VOC Omicron (BA.4/BA.5), SARS-CoV-1, and MERS-CoV, toxicity, and SI.

Compounds	Calu-3 Cells								
	SARS-CoV-2				SARS-CoV-1		MERS-CoV		Toxicity
	USA-WA1/2020		Omicron BA.4/BA.5						
	IC_50_ (nM) ^a^	SI	IC_50_ (nM) ^a^	SI	IC_50_ (nM) ^a^	SI	IC_50_ (nM) ^a^	SI	CC_50_ (µM) ^a^
NBCoV35	360 ± 35	>139	520 ± 27	>96	1247 ± 45	>40	1667 ± 225	>30	>50
NBCoV36	433 ± 49	>116	565 ± 79	>89	627 ± 58	>80	589 ± 117	>85	>50
NBCoV37	368 ± 45	>136	583 ± 35	>86	1000 ± 132	>50	877 ± 50	>57	>50
NBCoV63	120 ± 6	417	126 ± 5	397	133 ± 28	376	220 ± 30	227	50

^a^ The reported values represent the means ± standard deviations (n = 3).

**Table 6:** Antiviral activity of the NBCoV compounds in a single-cycle assay in 293T/ACE2 cells against control pseudovirus NL4-3^ΔEnv^Luc/A-MLV.

Compound	A-MLV IC_50_ (nM) ^a^
NBCoV35	1825 ± 25
NBCoV36	3300 ± 100
NBCoV37	1511 ± 88
NBCoV63	303 ± 29

^a^ The reported values represent the means ± standard deviations (n = 3).

**Table 7:** Antiviral activity of NBCoV63 against authentic viruses ^1^ in Calu-3 cells.

Virus	IC_50_ (µM) ^2^		CC_50_ (µM) ^3^
	NBCoV63	Remdesivir (Control)	
SARS-CoV-2 Hong Kong	2.04	3.44	>50
SARS-CoV-2 Delta (B.1.617.2)	9.27	12.5	
SARS-CoV-2 Omicron (BA.2.12.1)	5.45	2.82	
SARS-CoV-1 Urbani	3.98	1.08	
MERS-CoV	8.01	17.8	

^1^ The assay was performed by SRI International, Menlo Park, CA. ^2^ The reported values represent the means (n = 2) and the assay was repeated. ^3^ CC_50_ of both NBCoV63 and Remdesivir in Calu-3 cells was >50 µM.

**Table 8:** In vitro ADME profile of the most potent inhibitor, NBCoV63.

Assay Performed	In Vitro ADMET	Inhibitor
		NBCoV63
Solubility (µM)	Phosphate buffer, pH7.4	6.25
Caco-2 permeability (mean P_app_, ×10^−6^ cm/s)	A-to-B	0.00242
	B-to-A	0.19
	Efflux Ratio	42.3
Metabolic stability (human liver microsomes)	Cl_int_ (µL/min/mg protein)	3.4
	Half-life (min)	>180
Protein binding (human plasma)	% bound	>99.5
Cytochrome P450 inhibition, IC_50_ (µM)	CYP1A2 (a-Naphthoflavone)	>25
	CYP2B6 (Ticlopidine)	>25
	CYP2C8 (Quercetin)	>25
	CYP2C9 (Sulphaphenazole)	>25
	CYP2C19 (Ticlopidine)	>25
	CYP2D6 (Quinidine)	>25

## Data Availability

Data are available from the corresponding authors upon request.

## Supplementary Materials

The following supporting information can be downloaded at: https://www.mdpi.com/article/10.3390/v15041001/s1, Figure S1: A traditional plaque reduction assay was performed in 24 well plates.
